# Multimodal Management of Chronic Thromboembolic Pulmonary Hypertension: A Narrative Review of Treatment Selection and Sequencing

**DOI:** 10.3390/jcdd13090433

**Published:** 2026-09-03

**Authors:** Houda Gharsalli, Mohammed Alshahrani, Olfa Harbi, Nawal Alwadai, Abdalla M. Al-Asiri, Fatmah Alahmari, Amal Alqahtani, Najla Al-Jahash, Lobna Abbag, Saud Alqahtani, Khaled Abdulwahab Amer

**Affiliations:** 1 Pulmonology Department, Aseer Central Hospital, Abha 62523, Saudi Arabia; houdagharsalli@yahoo.fr (H.G.); malshahrani24@moh.gov.sa (M.A.); nana-1866@hotmail.com (N.A.); abokhogmah@hotmail.com (A.M.A.-A.); 2 Pulmonology Section, Medical Department, Imam Abdulrahman Al-Faisal Hospital, Riyadh 11461, Saudi Arabia; olfaharbichedly@yahoo.fr; 3 Department of Internal Medicine, Aseer Central Hospital, Abha 62523, Saudi Arabia; fatimah0alahmari@gmail.com (F.A.); amalalqahtani96@outlook.com (A.A.); najlajahash@gmail.com (N.A.-J.); fu.lubna@gmail.com (L.A.); dr.saudalqahtani.88@gmail.com (S.A.)

**Keywords:** chronic thromboembolic pulmonary hypertension, pulmonary endarterectomy, balloon pulmonary angioplasty, pulmonary hypertension-targeted therapy, multimodal management, anticoagulation, narrative review

## Abstract

Chronic thromboembolic pulmonary hypertension (CTEPH) is a treatable yet frequently underdiagnosed late complication of acute pulmonary embolism, in which incompletely resolved thromboemboli organise into fibrotic obstructions of the pulmonary arteries and, together with a secondary small-vessel arteriopathy affecting both obstructed and non-obstructed territories, raise pulmonary vascular resistance, impose right ventricular pressure overload, and—if left untreated—progress to right heart failure and death. Three established therapies address the disease at distinct anatomical levels: pulmonary endarterectomy, the potentially curative treatment of choice for surgically accessible disease; balloon pulmonary angioplasty, for distal inoperable lesions or residual disease; and pulmonary hypertension-targeted medical therapy, for the accompanying microvasculopathy. Their evaluation in randomised controlled trials and large registries has produced a multimodal paradigm in which these treatments are combined and sequenced according to operability and lesion distribution. This narrative review synthesises evidence published through January 2026 to accomplish the following: (i) summarise and critically appraise the contemporary evidence for each modality, distinguishing guideline-supported recommendations from observational, proof-of-concept, and expert-opinion-based practice; (ii) describe how treatments are selected, sequenced, and bridged in relation to operability and to residual or recurrent pulmonary hypertension after endarterectomy; and (iii) situate treatment within the broader chronic thromboembolic pulmonary disease spectrum and the case-finding pathway after pulmonary embolism. Rather than proposing a validated algorithm, it emphasises that, because each modality carries distinct indications and risks, decisions should be individualised through multidisciplinary assessment at expert centres, with early referral central to optimising outcomes.

## 1. Introduction

Chronic thromboembolic pulmonary hypertension (CTEPH) is an uncommon, potentially fatal complication that arises when pulmonary emboli fail to resolve completely after at least three months of effective anticoagulation [1,2]. The persistent thromboembolic material organises into fibrotic webs and stenoses that obstruct the proximal and distal pulmonary arteries; in parallel, a secondary small-vessel arteriopathy develops—not only in the non-obstructed territories exposed to high flow and pressure, but also, through a distinct mechanism, within the obstructed segments, where the systemic bronchial collateral circulation is thought to contribute—adding further to the haemodynamic burden and to the development of pulmonary hypertension (PH) [3]. CTEPH is reported in approximately 1–3.8% of patients within two years of an acute pulmonary embolism [4,5] and is categorised as Group 4 in the clinical classification of PH [6].

Left untreated, CTEPH is progressive and may culminate in right heart failure and death [7,8], a course made more complex by the frequent coexistence of proximal and distal lesions within the same patient [9]. Early recognition and timely referral are therefore essential [3]. Three therapeutic modalities target the disease at its different anatomical levels: pulmonary endarterectomy (PEA) addresses proximal, surgically accessible obstructions; balloon pulmonary angioplasty (BPA) treats more distal lesions; and PH-targeted medical therapy acts on the accompanying microvasculopathy. Because these modalities can be combined in various sequences, they form the basis of a multimodal therapeutic strategy that should be planned on a case-by-case basis through multidisciplinary discussion at experienced CTEPH centres. Building on the 2022 ESC/ERS guidelines and on randomised, registry, and proof-of-concept data published through 2026, this review consolidates the current evidence for each modality and frames a practical, lesion-based approach to support multimodal treatment selection.

Several authoritative syntheses and guidelines have charted the emergence of multimodal CTEPH care [10,11,12]. What is less consistently translated to the bedside is an explicit, evidence-graded account of how the three modalities are chosen, ordered, and bridged in the individual patient, and of how confident clinicians can be in each step. This narrative review is organised around that practical question; rather than proposing a new validated algorithm, it sets out to distinguish what is guideline-supported from what rests on observational series, small proof-of-concept studies, or expert-centre practice. We address four questions in particular: (i) how operability is determined, and why it is a multidisciplinary judgement rather than a threshold-based one; (ii) how PEA, BPA, and medical therapy compare and are combined; (iii) how residual or recurrent PH after PEA should be defined and managed; and (iv) how anticoagulation and the wider chronic thromboembolic pulmonary disease spectrum fit into the therapeutic framework. Because CTEPH lies at the interface of venous thromboembolism, the pulmonary circulation, and the right ventricle, it is of direct relevance to the cardiovascular community: cardiologists increasingly perform balloon pulmonary angioplasty, interpret the right ventricular consequences of pulmonary vascular obstruction, and oversee the post-embolic follow-up through which most cases come to light. We therefore link the treatment evidence to the upstream diagnostic pathway and to the expanding concept of chronic thromboembolic pulmonary disease, and we conclude by setting out the principal evidence gaps and the trials designed to close them.

## 2. Aim and Search Strategy

This is a non-systematic narrative review; it was not conducted or reported according to PRISMA, and no formal risk-of-bias appraisal or quantitative synthesis was undertaken. Its aim is to summarise and critically appraise the contemporary evidence underpinning each treatment modality for CTEPH and to illustrate how a multimodal approach influences disease management. A literature search was conducted in PubMed/MEDLINE, Scopus, ScienceDirect, and SpringerLink from database inception to January 2026, combining controlled vocabulary and free-text terms: (“chronic thromboembolic pulmonary hypertension” OR “CTEPH”) AND (“multimodal treatment” OR “combined therapy” OR “pulmonary endarterectomy” OR “balloon pulmonary angioplasty” OR “medical therapy” OR “anticoagulant therapy”). The search was limited to full-text articles published in English. Titles and abstracts were screened by the authors for relevance, and the reference lists of key reviews and guidelines were hand-searched to identify further sources. Priority was given, in descending order of evidential weight, to current international guidelines and clinical consensus statements; randomised controlled trials and their meta-analyses; large multicentre registries; and finally to smaller observational cohorts, proof-of-concept studies, and case series, which were included where they addressed questions not covered by higher-level evidence. Throughout the text, tables, and figures, we have sought to indicate the nature and strength of the evidence supporting each statement, distinguishing guideline-supported recommendations from observational data, proof-of-concept experience, and expert opinion. Because study selection and interpretation were not performed in duplicate against pre-specified criteria, the synthesis necessarily reflects a degree of author judgement, which we have aimed to make transparent.

## 3. Therapeutic Options in CTEPH

**Figure 1:**
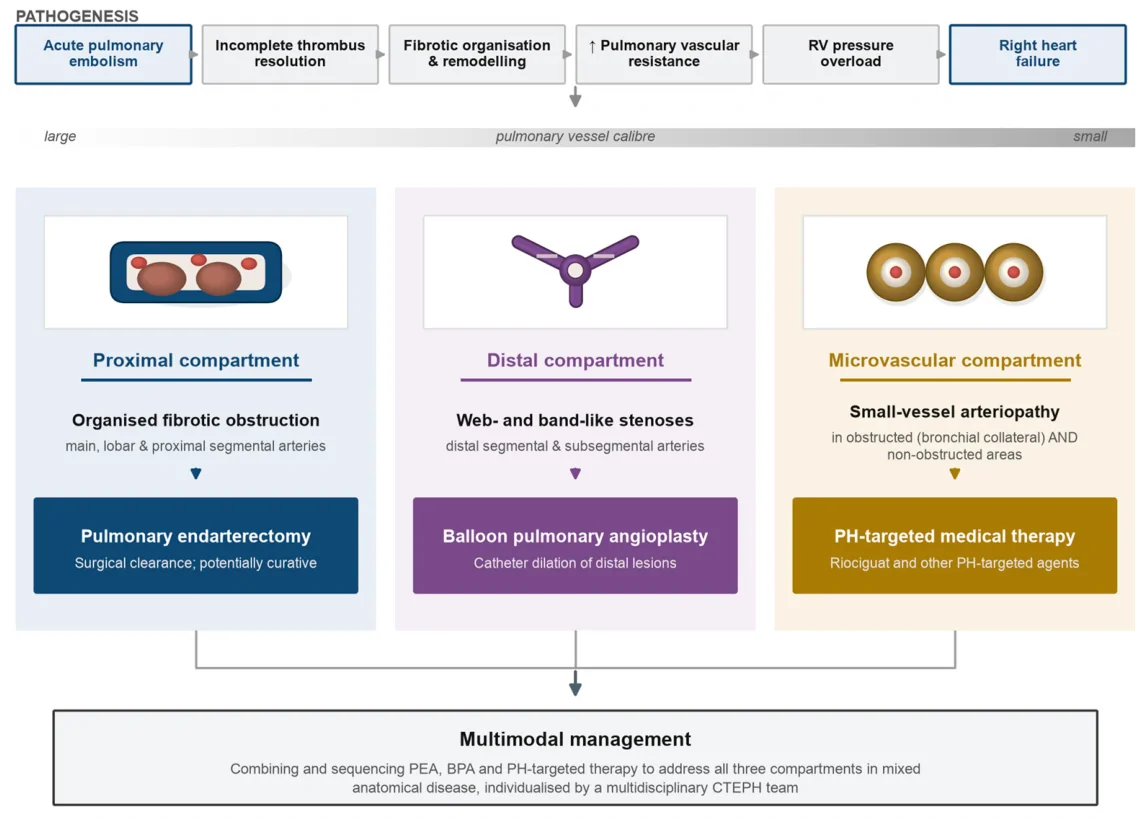
The three-compartment model of CTEPH and the rationale for multimodal therapy. Unresolved thromboemboli organise into fibrotic obstructions of the proximal and distal pulmonary arteries, while a secondary small-vessel arteriopathy develops in non-obstructed territories; together, these raise pulmonary vascular resistance and drive right ventricular pressure overload. Each compartment is addressed by a complementary therapy—pulmonary endarterectomy for proximal obstruction, balloon pulmonary angioplasty for distal stenoses, and PH-targeted medical therapy for the microvasculopathy—providing the rationale for combining and sequencing modalities in multimodal management. BPA, balloon pulmonary angioplasty; CTEPH, chronic thromboembolic pulmonary hypertension; PEA, pulmonary endarterectomy; PH, pulmonary hypertension; RV, right ventricular.

The overarching goals of CTEPH treatment are to relieve symptoms, to improve exercise capacity and quality of life, and to normalise—or near-normalise—resting haemodynamics [10]. Three evidence-based therapeutic options target the distinct anatomical lesions of the disease. This reflects the three-compartment nature of CTEPH, in which proximal fibrotic obstruction, distal web- and band-like stenoses, and a small-vessel arteriopathy of the non-obstructed pulmonary vasculature coexist in varying proportions and are addressed by complementary therapies (Figure 1):

- PEA is the treatment of choice and a potentially curative option when the disease is surgically accessible [7,10,13,14].
- BPA is an established catheter-based revascularisation technique for inoperable disease or for residual/recurrent PH after PEA; global expertise and procedural safety have improved considerably [11,15,16,17,18,19,20,21,22,23,24,25].
- PH-targeted therapy is central to the management of microvasculopathy and is used in inoperable disease, in residual/recurrent PH, and for selective preoperative optimisation before PEA [10,12,26,27,28,29,30,31,32,33,34,35,36,37].

Table 1 summarises the indications, factors precluding eligibility, principal complications, and expected outcomes of PEA and BPA.

## 4. Patient Selection, Operability, and Risk Stratification

The first and most consequential decision in CTEPH management is whether a patient is operable, because this single judgement determines the entire treatment pathway [10,12]. Operability is not a binary radiological property but a multidimensional assessment that integrates the anatomical accessibility of the obstructive lesions, the match between the measured pulmonary vascular resistance and the visible obstructive burden, patient comorbidity, and overall surgical risk; it should be established by an experienced multidisciplinary team at a high-volume centre, and judgements of operability are known to differ substantially between centres [10,41].

Lesion distribution is the principal anatomical determinant: surgically accessible disease of the main, lobar, or proximal segmental arteries favours PEA, whereas predominantly distal disease favours BPA [7,10]. Imaging—ventilation–perfusion scintigraphy, computed tomographic pulmonary angiography, and selective digital subtraction angiography, increasingly complemented by cone-beam computed tomography—defines this distribution and guides the choice and planning of intervention [10,12]. The characteristic findings are mismatched segmental or subsegmental perfusion defects on ventilation–perfusion (V/Q) scintigraphy, and eccentric wall-adherent thrombus, intraluminal webs and bands, abrupt vessel narrowing or cut-offs, poststenotic dilatation, and enlarged bronchial collateral arteries on computed tomographic pulmonary angiography, with pouch defects, web-like and ring-like stenoses, and complete obstructions delineated on selective digital subtraction angiography; recognition of these patterns, rather than any single modality, underpins both diagnosis and procedural planning [10,12]. Non-invasive imaging also underpins risk stratification: transthoracic echocardiography is the first-line tool for detecting PH and assessing right ventricular size, function, and right ventricular–arterial coupling (for example, the tricuspid annular plane systolic excursion to systolic PA pressure ratio) [42], while cardiac magnetic resonance provides the reference-standard, reproducible assessment of right ventricular volumes, mass, and ejection fraction and is increasingly used for prognostication and follow-up. A preoperative pulmonary vascular resistance that is disproportionately high relative to the visible obstruction (a PVR–obstruction mismatch) signals a larger small-vessel component and predicts a greater risk of persistent pulmonary hypertension and worse outcomes after surgery [41,43]. In this specific situation—a PVR–imaging obstruction discordance, particularly when accompanied by right ventricular failure—preoperative medical optimisation of the right ventricle before PEA is undertaken at some expert centres; this is a reasonable, physiologically motivated practice but rests on limited published support and expert opinion rather than randomised evidence, in contrast to the randomised data underpinning riociguat pre-treatment before BPA (see Section 7.2 and Section 7.4). Haemodynamic assessment during exercise can unmask a disproportionate rise in pulmonary pressures and provide additional functional information for selection and for evaluating the response to intervention [44,45].

Comorbidities such as advanced age, coronary artery disease, left ventricular dysfunction, and severe lung or renal disease raise operative risk and act as risk modifiers within the operability assessment; they may shift selected patients toward BPA or medical therapy even when the lesions are technically accessible, but—with the exception of severe intrinsic parenchymal lung disease, the one true contraindication to PEA—they rarely exclude a patient outright [7,10]. Operability determination also depends on how lesions are classified. The original University of California San Diego (UCSD) surgical classification (levels I–IV) described the most proximal level of disease encountered at operation; it has since been refined into an updated intraoperative classification (levels I–IV, including a level 0 for no identifiable disease and subdivisions of level I) that better reflects contemporary practice and correlates with haemodynamics and outcome. Familiarity with both the older and newer schemes is useful when interpreting the literature and communicating within the team. Because operability and risk are dynamic rather than fixed, a patient judged inoperable or high-risk at one centre may become a candidate after medical optimisation or when reassessed at a more experienced centre; assessments differ substantially between centres, and what appears inoperable to a less experienced surgeon may be operable disease to an experienced one. A second opinion at a high-volume centre should therefore be sought in difficult cases, and referral to an expert centre before disease is declared inoperable is essential [9,10,41]. These judgements are best made by a dedicated multidisciplinary CTEPH team—comprising a PEA surgeon, a BPA interventionalist, a PH physician, and a radiologist with expertise in pulmonary vascular imaging—meeting regularly, with joint review of ventilation–perfusion, computed tomographic, and angiographic imaging alongside haemodynamics and comorbidity [10,41]. The principal factors that guide operability and treatment selection are summarised in Table 2.

### Procedural Techniques and Management of Complications

Both procedures are technically demanding and are performed at high-volume centres, and an appreciation of their technique and complications informs treatment selection. PEA is performed through a median sternotomy on cardiopulmonary bypass with deep hypothermic circulatory arrest to achieve the bloodless field required for a complete bilateral endarterectomy; a true dissection plane must be established within the media. The principal early complications are reperfusion lung injury, residual pulmonary hypertension, and, less commonly, neurological events; refractory right ventricular failure or severe reperfusion injury may require venoarterial or venovenous extracorporeal membrane oxygenation (ECMO) as a bridge to recovery, and pre-planned ECMO availability is part of the safety framework at expert centres. BPA is a staged procedure in which pulmonary arterial webs and stenoses are dilated over several sessions, deliberately treating a limited number of segments per session to reduce the risk of reperfusion pulmonary oedema. Its characteristic complications are vascular injury and haemoptysis; their management has matured and now includes prolonged low-pressure balloon inflation to seal a leak, reversal of heparin with protamine, selective coil or particle embolisation of a bleeding vessel, and, for frank perforation, deployment of a covered stent, with temporary ECMO support reserved for the rare catastrophic event [15,19,46,47]. This procedural safety net, together with refinements in patient and lesion selection, largely accounts for the improving safety profile of BPA reported across contemporary registries.

## 5. Pulmonary Endarterectomy Versus Balloon Pulmonary Angioplasty

No completed randomised trial has yet directly compared PEA and BPA in patients whose lesions are amenable to either procedure, and the two modalities are applied to systematically different populations. PEA is a single, potentially curative operation carrying an upfront operative risk, whereas BPA is a staged, lower-risk procedure typically delivered over several sessions; the patients selected for each differ in age, comorbidity, and lesion distribution. For these reasons, the available pooled data—derived almost entirely from heterogeneous single-arm observational cohorts spanning different eras and centres—can describe each modality but cannot validly rank one against the other, and any apparent survival difference largely reflects between-group differences in baseline risk rather than a true superiority of either technique. We therefore do not tabulate indirect comparisons here. This important evidence gap is being addressed prospectively by the multinational GO-CTEPH trial (NCT05110066), which is randomising patients eligible for both modalities and will use the change in PVR at 4 and 12 months after the procedure as its primary endpoint, testing whether BPA is non-inferior to PEA [48]. Until such head-to-head data are available, the choice between PEA and BPA rests on multidisciplinary assessment of lesion accessibility, haemodynamics, comorbidity, and patient preference rather than on any ranked comparison of outcomes.

## 6. PH-Targeted Medical Therapy

**Figure 2:**
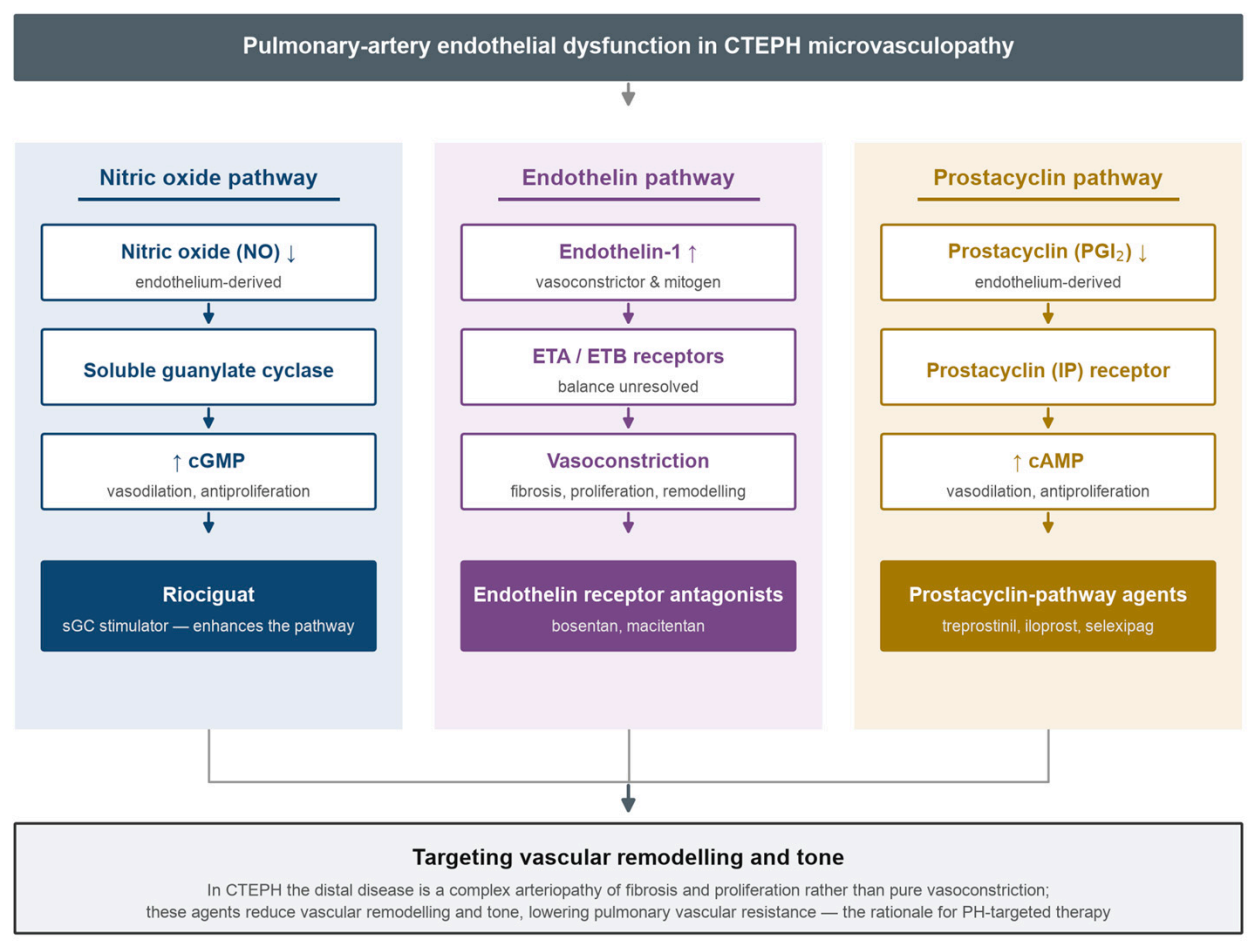
Mechanisms of action of pulmonary hypertension-targeted drug classes in CTEPH. Endothelial dysfunction reduces the bioavailability of the vasodilators nitric oxide and prostacyclin and increases production of the vasoconstrictor endothelin-1. Riociguat stimulates soluble guanylate cyclase to raise cyclic GMP; endothelin receptor antagonists (bosentan, macitentan) block the ET_A_ and ET_B_ receptors; and prostacyclin-pathway agents (treprostinil, iloprost, selexipag) activate the prostacyclin (IP) receptor to raise cyclic AMP. Although these pathways were historically termed vasodilator targets, in CTEPH the distal small-vessel disease is a complex arteriopathy of fibrosis and proliferation rather than a predominantly vasoconstrictive process; accordingly, we use the term PH-targeted therapy, and the net effect of these agents is better understood as reduced vascular remodelling and tone with a consequent fall in pulmonary vascular resistance. cAMP, cyclic adenosine monophosphate; cGMP, cyclic guanosine monophosphate; CTEPH, chronic thromboembolic pulmonary hypertension; ET, endothelin; IP, prostacyclin; PGI2, prostacyclin; sGC, soluble guanylate cyclase.

Even after the mechanical obstruction has been relieved surgically or percutaneously, distal small-vessel remodelling and microvasculopathy may persist. PH-targeted drugs therefore have an increasingly well-defined role in CTEPH, particularly in inoperable disease, in residual or recurrent PH after PEA, and for peri-interventional optimisation [12,26]. The pivotal randomised trials of medical therapy and of BPA versus medical therapy are summarised in Table 3. The approved and investigational agents act on three signalling pathways that govern pulmonary vascular tone and remodelling (Figure 2).

### 6.1. Soluble Guanylate Cyclase Stimulator (Riociguat)

Riociguat is the most thoroughly studied agent in CTEPH. In the CHEST-1 trial and its CHEST-2 long-term extension, riociguat produced clinically meaningful gains in 6 min walk distance (6MWD) together with improvements in PVR, mean pulmonary artery pressure (mPAP), functional class, and N-terminal pro-B-type natriuretic peptide (NT-proBNP). On the strength of these data, it became the first drug approved for inoperable or residual CTEPH and carries a Class I, Level B recommendation in the 2022 ESC/ERS guidelines [12,36,37].

### 6.2. Endothelin Receptor Antagonists

Endothelin-1 is a potent vasoconstrictor and mitogen acting through ET_A_ and ET_B_ receptors; which of the two is preferable to block in CTEPH remains unresolved, and this uncertainty is reflected in the mixed trial results. Bosentan, a dual ET_A_/ET_B_ antagonist evaluated in the BENEFiT trial, improved PVR but did not significantly change 6MWD [29]. The evidence for macitentan illustrates how dose, population, and trial design shape conclusions. In the phase 2 MERIT-1 trial, macitentan 10 mg significantly improved PVR and also 6MWD, NT-proBNP, and cardiac output in inoperable CTEPH; because many participants received background phosphodiesterase-5 inhibitors or prostanoids, this trial also provided early evidence for combination therapy, although concurrent riociguat was not permitted [31] (the original 2017 report was retracted and republished in 2024 following correction of statistical errors; the corrected analysis preserved the principal PVR finding, and the republished version is cited here [31]). The subsequent phase 3 MACiTEPH programme tested a higher 75 mg dose in a broader inoperable or persistent/recurrent population and was stopped early for futility after a pre-planned interim analysis, with no treatment effect on 6MWD [49]. The contrast between a positive phase 2 and a negative phase 3 result—differing in dose, endpoint, and population, and against a background of contemporary multimodal care—cautions strongly against extrapolating early haemodynamic signals to clinical benefit. An ambrisentan programme in inoperable CTEPH was closed prematurely owing to slow enrolment [33].

### 6.3. Prostacyclin Pathway Agents

In the phase 3 CTREPH trial, higher-dose subcutaneous treprostinil improved 6MWD and PVR relative to a low-dose comparator, supporting its use in severe inoperable CTEPH and in residual PH after PEA; long-term tolerability and sustained benefit were confirmed in the open-label extension [30,50]. It should be noted, however, that parenteral prostanoid therapy in CTEPH is largely supported by this single randomised comparison together with open-label experience rather than by placebo-controlled outcome data. Inhaled iloprost improved PVR and 6MWD within the CTEPH subset of an earlier, mixed-aetiology PH trial [52]. The selective prostacyclin (IP) receptor agonist selexipag has followed a divergent regulatory path that illustrates the hazard of generalising across populations: in a dedicated Japanese randomised trial, it significantly reduced PVR—leading to its approval for inoperable or persistent/recurrent CTEPH in Japan—although mPAP and 6MWD were not significantly changed and many participants were receiving background riociguat and had undergone prior BPA [32]; by contrast, the larger global SELECT trial was terminated for futility, having shown no PVR benefit over placebo [35]. Selexipag is therefore approved in one jurisdiction on the strength of a positive dedicated trial yet unsupported by the global programme.

### 6.4. BPA Versus PH-Targeted Medical Therapy

Two randomised trials established the superiority of BPA over medical therapy alone for haemodynamic improvement in inoperable CTEPH. In the Japanese MR-BPA trial, the 12-month reduction in mPAP was −16.3 mmHg with BPA compared with −7.0 mmHg with riociguat [51]. In the French RACE trial, the percentage reduction in PVR at 26 weeks favoured BPA over riociguat, although both treatments improved haemodynamics [21]. Importantly, in RACE, pre-treatment with riociguat reduced BPA-related serious adverse events (14% versus 42%), providing a rationale for pharmacological pre-optimisation in patients with high baseline PVR [21].

Taken together, the medical-therapy evidence base is uneven, and its interpretation depends heavily on trial design and endpoint. Riociguat remains the only agent with robust, sustained randomised effects on both exercise capacity and haemodynamics and the only one carrying a Class I recommendation and broad regulatory approval in CTEPH. Among the endothelin receptor antagonists, bosentan improved PVR but not walking distance, and the contrasting phase 2 and phase 3 macitentan results leave the class unproven for clinical outcomes. In the prostacyclin pathway, subcutaneous treprostinil is supported by a single randomised dose-comparison and open-label experience in severe disease, whereas selexipag is approved only in Japan and was negative in the global SELECT trial. Cross-trial comparison is further constrained by the predominance of PVR-based primary endpoints—often expressed as a ratio or percentage of baseline—and by heterogeneous background therapy. Two practical conclusions follow: riociguat is the medical therapy of choice for inoperable or residual/recurrent CTEPH, and the evidentiary bar for adopting other agents—particularly outside severe inoperable disease—remains high, underscoring the need for trials powered for clinical rather than purely haemodynamic outcomes.

## 7. The Multimodal Approach in CTEPH

**Figure 3:**
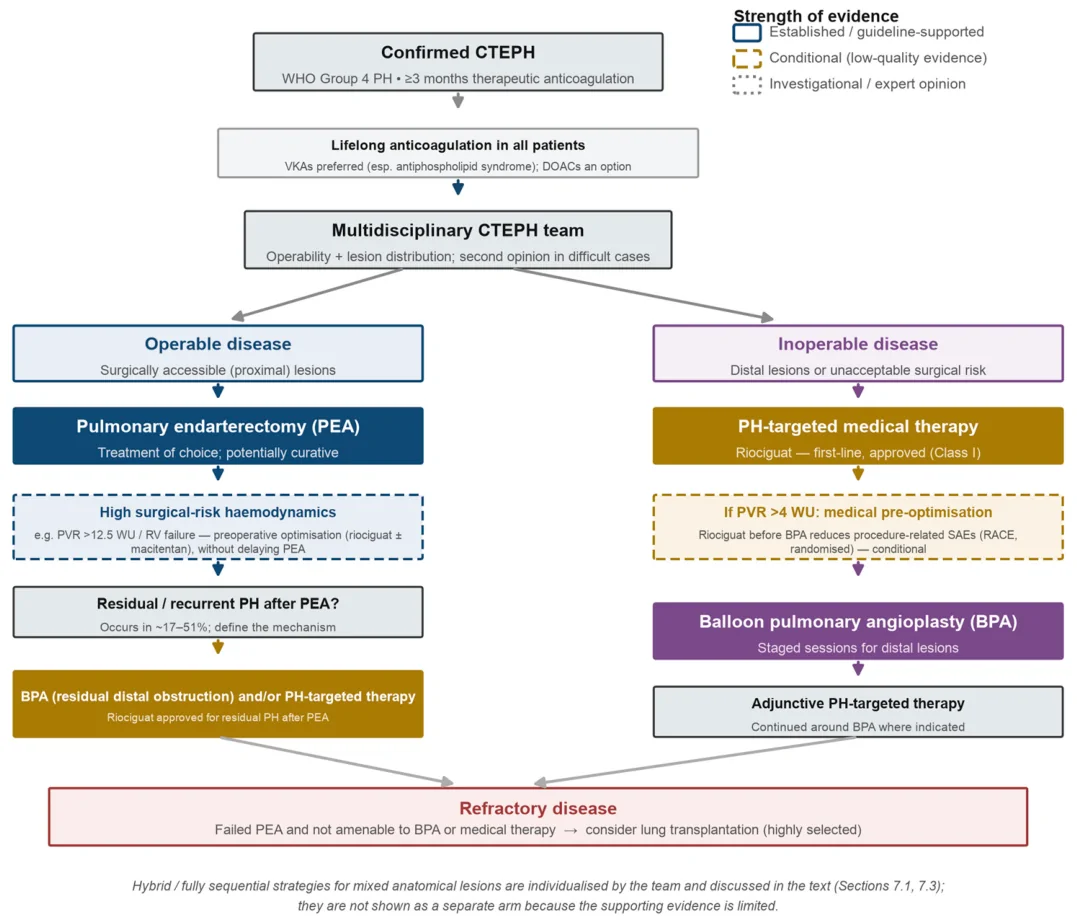
Evidence-graded framework for the multimodal management of CTEPH. After multidisciplinary operability assessment, patients follow one of two principal arms—operable disease directed to pulmonary endarterectomy, and inoperable disease directed to balloon pulmonary angioplasty and/or PH-targeted medical therapy—with the multidisciplinary team integrating lesion distribution, haemodynamics, comorbidity, and patient preference. Steps are shaded by strength of evidence: established, guideline-supported recommendations are distinguished from conditional strategies based on low-quality evidence and from investigational or expert-opinion-based options. Hybrid and fully sequential strategies for mixed anatomical lesions are not presented as a separate arm because the supporting evidence is limited; they are considered on an individual basis by the team (see Section 7.1 and Section 7.3). BPA, balloon pulmonary angioplasty; CTEPH, chronic thromboembolic pulmonary hypertension; PEA, pulmonary endarterectomy; PH, pulmonary hypertension; PVR, pulmonary vascular resistance; WU, Wood units.

CTEPH management has advanced substantially over the past decade, and the systematic evaluation of treatment combinations has given rise to the concept of multimodal management. This strategy combines at least two of the three modalities to target the different vascular components of the disease [10,12]. It is important to be explicit about the strength of evidence behind each combination: the superiority of BPA over medical therapy in inoperable disease and the value of riociguat pre-treatment before BPA rest on randomised trials, whereas most sequential and hybrid strategies are supported only by small observational series, proof-of-concept studies, or expert-centre experience and should be regarded as conditional or investigational. Accordingly, the algorithm in Figure 3 is deliberately confined to the two evidence-based arms—operable disease directed to PEA and inoperable disease directed to BPA and/or PH-targeted therapy—with the multidisciplinary team weighing comorbidity, haemodynamics, and patient preference; the more exploratory hybrid and fully sequential approaches are discussed in the text below as creative solutions to difficult individual problems rather than as established, equally weighted options. Multimodal treatment can benefit selected patients but should always be planned by a multidisciplinary CTEPH team.

### 7.1. Combining PEA with BPA

Several reports—observational series and small cohorts rather than randomised trials—now document the safe combination of PEA and BPA across different sequences: PEA followed by elective BPA for residual distal disease; BPA followed by PEA; and hybrid approaches in which PEA is performed on the operable side and BPA on the contralateral, inoperable lung [23,41,53,54,55,56,57]. This evidence is of low quality and derived from highly selected patients at expert centres, so these combinations should be regarded as conditional, individualised strategies rather than established recommendations. Urgent BPA in the immediate aftermath of PEA is generally avoided because of haemodynamic instability [27]. Conversely, BPA performed before PEA may disrupt the endarterectomy plane and complicate surgery in dilated segments; the timing of each procedure must therefore be individualised by centres experienced in both techniques [56,57].

### 7.2. PH-Targeted Therapy as a Bridge to PEA

Routine PH-targeted therapy as a bridge to PEA has no proven benefit and risks delaying curative surgery [9,58]. In patients with very high-risk haemodynamics, however—for example, a PVR above 12.5 Wood units or a low cardiac output, particularly with right ventricular failure—preoperative pharmacological optimisation of the right ventricle may be considered, and riociguat, with or without macitentan, can be a reasonable option to reduce perioperative risk, provided it does not delay referral to an expert PEA centre [9,43,59]. It must be emphasised that the strength of evidence here differs fundamentally from that for pre-BPA optimisation: whereas riociguat before BPA is supported by a randomised trial (RACE; see Section 7.4), preoperative medical optimisation before PEA rests on physiological reasoning and limited observational experience rather than randomised data, and is best regarded as an expert-opinion-based, conditional practice.

### 7.3. Sequential Medical Therapy and BPA as a Bridge to PEA

A recent proof-of-concept study enrolled only nine carefully selected patients with mixed (proximal and distal) lesions and very high preoperative PVR (>800 dyn·s·cm^−5^) and applied a fully sequential strategy combining medical therapy, BPA, and PEA [54]. PH-targeted therapy was started before interventional treatment to improve the safety of BPA, after which BPA was used to optimise preoperative haemodynamics and to allow PEA to be confined to one side, shortening the duration of circulatory arrest and cardiopulmonary bypass. The combination improved pulmonary haemodynamics, although the response varied between individuals. This is an illustrative, hypothesis-generating experience from a single expert centre; given the very small, highly selected sample, it should be interpreted as a creative solution to an exceptionally difficult problem rather than as a validated pathway, and it carries far less weight than the randomised evidence supporting the individual modalities [54].

### 7.4. Bridging Therapy Before BPA

The RACE trial provides randomised evidence that pre-treating inoperable, high-resistance patients with riociguat before BPA reduces procedure-related serious adverse events and yields additive haemodynamic benefit [21]. The 2022 ESC/ERS guidelines suggest considering medical therapy before BPA when PVR exceeds 4 Wood units, albeit on the basis of low-quality evidence [12]. The ongoing IMPACT-CTEPH trial (NCT04780932) is testing whether dual oral therapy (riociguat plus macitentan) is more effective than riociguat monotherapy—with respect to PVR reduction, exercise capacity, and WHO functional class—for pre-BPA optimisation in inoperable CTEPH [60]. Complementing these pre-procedural strategies, the phase 4 THERAPY-HYBRID-BPA trial found that continuing riociguat after BPA attenuated the deterioration of exercise capacity without additional safety concerns, supporting a role for medical therapy around the time of the BPA procedure [61].

### 7.5. BPA and PH-Targeted Therapy in Residual or Recurrent PH

Residual or recurrent PH after PEA is one of the central clinical problems in CTEPH, and precise terminology matters. Persistent (residual) PH denotes pulmonary hypertension that never resolves after surgery, typically identified at early post-operative or 3–6-month reassessment, and usually reflects either residual distal obstruction that was not surgically accessible or a fixed small-vessel arteriopathy (microvasculopathy). Recurrent PH describes the later return of pulmonary hypertension after an initial good haemodynamic result, which may follow re-thrombosis, progression of distal disease, or progressive microvasculopathy. Distinguishing these mechanisms is clinically important because residual distal obstruction is potentially amenable to BPA, whereas predominant microvasculopathy is a target for PH-targeted medical therapy. The literature is complicated by heterogeneous definitions: residual PH has been variously defined as an mPAP ≥ 25 mmHg or ≥30 mmHg and/or an elevated PVR, and the recently proposed lower haemodynamic threshold for PH (mPAP > 20–21 mmHg) has not yet been adopted in the residual-PH literature, so reported frequencies—approximately 17–51% of patients—vary widely and are difficult to compare across studies [18,43,59,62]. This variability is not merely semantic: the prognostic threshold at which residual PH begins to affect survival appears lower than the historical definitions, so that patients with apparently “mild” residual elevation may still be at risk, which argues for a consistent, contemporary definition and for structured haemodynamic reassessment (by right heart catheterisation) at a defined interval, commonly 3–6 and 12 months after PEA. Management is guided by mechanism: BPA can effectively relieve the distal obstructions that persist after surgery, with multiple studies reporting improved haemodynamics, symptoms, and exercise capacity, and can reverse right ventricular remodelling and dysfunction [53,62,63,64]; riociguat is approved for residual PH after PEA and improves both exercise capacity and PVR [12,36,37]; and long-term subcutaneous treprostinil has shown sustained benefit and tolerability in this setting in the CTREPH open-label extension [50]. Beyond resting haemodynamics, exercise right heart catheterisation and cardiopulmonary exercise testing after PEA or BPA can reveal persistent exercise-induced pulmonary hypertension and impaired right ventricular–arterial coupling in patients whose resting values have normalised, helping to explain residual symptoms and to guide further intervention.

## 8. Other Therapeutic Considerations

Anticoagulation is prescribed lifelong for all patients with CTEPH, reflecting the thrombotic basis of the disease, although no dedicated randomised trial has defined the optimal agent [12]. Vitamin K antagonists (VKAs) have historically been the most widely used class. Direct oral anticoagulants (DOACs) are increasingly prescribed for convenience and because they avoid routine monitoring, and retrospective analyses suggest broadly comparable bleeding rates and survival relative to VKAs; however, a large multicentre cohort of patients undergoing PEA found a significantly higher rate of recurrent venous thromboembolism with DOACs than with VKAs despite similar major-bleeding rates [65], and DOAC use has also been associated with acute or subacute thrombus identified at the time of PEA [66]. Histological examination of PEA specimens frequently shows layered, organised, and partially recanalised thrombus of different ages, consistent with recurrent or in situ thrombosis and reinforcing the rationale for uninterrupted, effective anticoagulation. Perioperative management requires bridging around PEA, and monitored VKA therapy allows more predictable reversal than some DOACs. On balance, although DOACs are practical for many patients, the signal toward recurrent thromboembolism and peri-operative thrombus suggests that VKAs remain a reasonable default for patients who can be reliably monitored and are preferred where a hypercoagulable state is identified, pending dedicated randomised data.

A specific and important consideration is antiphospholipid syndrome (APS), an autoimmune thrombophilia that is over-represented among patients with CTEPH and should be actively sought, particularly in unprovoked or recurrent disease. Its identification changes management in two ways. First, DOACs are contraindicated in high-risk (triple-positive) APS: the randomised TRAPS trial was stopped early because of an excess of thromboembolic and bleeding events in patients assigned to rivaroxaban rather than warfarin [67], so dose-adjusted VKA therapy remains the standard, and APS is a further reason to favour VKAs over DOACs in CTEPH. Second, patients with APS may require additional immunomodulatory therapy directed at the underlying autoimmune process, managed jointly with rheumatology. Screening for antiphospholipid antibodies should therefore form part of the diagnostic work-up, and a positive result should prompt both a VKA-based anticoagulation strategy and multidisciplinary input.

Lung transplantation is now rarely indicated in CTEPH and is reserved for highly selected patients with failed PEA or with inoperable disease not amenable to BPA or medical therapy; outcome data are limited and some series report high morbidity and mortality [66]. Pulmonary artery denervation is a novel technique under investigation in PH: in residual PH after PEA, one study comparing denervation with riociguat reported a substantial reduction in PVR and an improvement in 6MWD, although this remains an investigational, expert-opinion–level option [68].

Finally, the therapeutic framework increasingly extends to symptomatic chronic thromboembolic pulmonary disease (CTEPD) without resting pulmonary hypertension—patients with persistent, imaging-confirmed chronic thromboembolic obstruction and exertional symptoms but a resting mPAP ≤ 20 mmHg. These patients are managed within the same expert-centre pathway as CTEPH: lifelong anticoagulation is continued, and selected patients with accessible disease and exercise limitation, exercise-induced pulmonary hypertension, or impaired right ventricular–arterial coupling may benefit from PEA or BPA to relieve symptoms, although the evidence base is smaller than for overt CTEPH and treatment decisions rest on multidisciplinary judgement [3,10]. Recognising CTEPD as part of the same disease spectrum ensures that symptomatic patients are not denied assessment simply because they fall below the resting haemodynamic threshold for pulmonary hypertension.

## 9. Future Directions and Unmet Needs

Despite substantial progress, several questions central to multimodal management remain unresolved. No completed randomised trial has directly compared pulmonary endarterectomy with balloon pulmonary angioplasty in patients eligible for either approach, and the GO-CTEPH trial is awaited to inform this choice [48]. The optimal timing and sequencing of combined treatment—when to bridge with medical therapy, whether to intervene percutaneously before or after surgery, and how best to manage the contralateral lung in mixed disease—still rest largely on observational series and expert consensus [11,54,56]. The value of peri-procedural and adjunctive medical therapy is being defined prospectively by trials such as IMPACT-CTEPH and THERAPY-HYBRID-BPA [60,61], yet the broader pharmacological pipeline has narrowed: beyond riociguat, successive phase 3 programmes have been negative or halted [35,49], so future studies will need to be powered for clinical rather than purely haemodynamic endpoints. A consistent, contemporary definition of residual pulmonary hypertension—aligned with the current haemodynamic threshold for pulmonary hypertension—is likewise needed if studies are to be compared [59].

**Figure 4:**
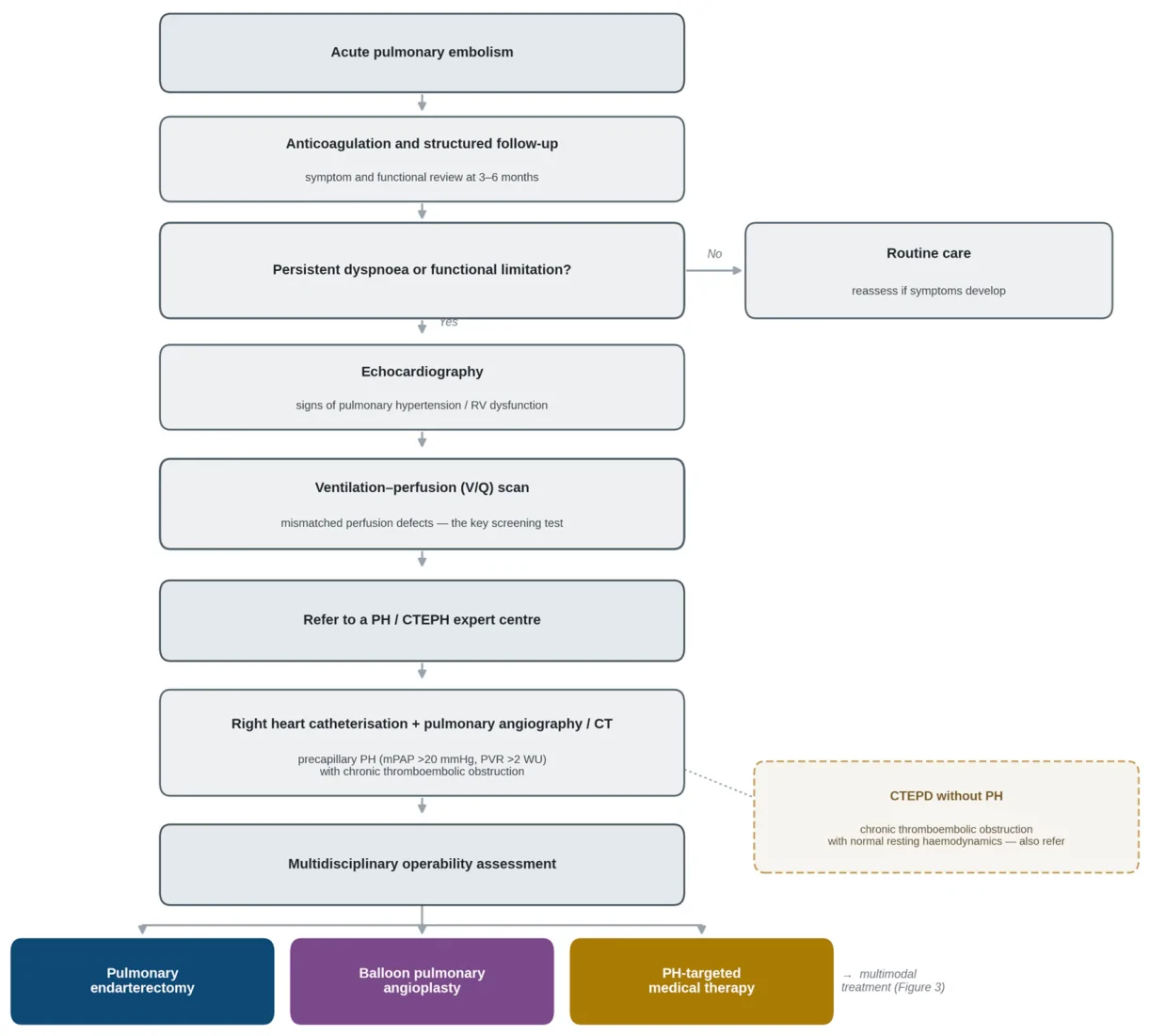
Proposed case-finding pathway for chronic thromboembolic pulmonary hypertension (CTEPH) after acute pulmonary embolism. Structured, symptom-based follow-up after pulmonary embolism identifies patients for non-invasive screening with echocardiography and ventilation–perfusion scanning; those with mismatched perfusion defects are referred to an expert centre for confirmation by right heart catheterisation and pulmonary angiography or computed tomography, after which multidisciplinary assessment of operability directs selection among pulmonary endarterectomy, balloon pulmonary angioplasty, and PH-targeted medical therapy (see Figure 3). Symptomatic chronic thromboembolic pulmonary disease without resting pulmonary hypertension (CTEPD) warrants the same referral. CT, computed tomography; CTEPD, chronic thromboembolic pulmonary disease; mPAP, mean pulmonary arterial pressure; PH, pulmonary hypertension; PVR, pulmonary vascular resistance; RV, right ventricular; V/Q, ventilation–perfusion; WU, Wood units.

Upstream of treatment, earlier diagnosis remains the single greatest unmet need. CTEPH develops in approximately 2–3% of survivors of acute pulmonary embolism, with right ventricular dysfunction at presentation, unprovoked embolism, and recurrent venous thromboembolism identifying higher-risk patients [69]; yet diagnosis is often delayed, and structured post-embolism follow-up programmes increase detection and capture disease at a less advanced, more treatable stage [70,71]; a pragmatic pathway from acute pulmonary embolism through case-finding to multidisciplinary treatment selection is summarised in Figure 4. Embedding simple, symptom-led screening into routine aftercare following pulmonary embolism, and clarifying when and how to treat the wider chronic thromboembolic pulmonary disease spectrum—including patients with chronic thromboembolic disease without resting pulmonary hypertension—are priorities for the years ahead [10]. Equitable and timely access to expert centres, the integration of advanced imaging and biomarkers into operability assessment, and the routine capture of patient-centred outcomes such as exercise capacity and quality of life will be essential if the expanding therapeutic armamentarium is to translate into longer and better lives for patients with this potentially curable disease.

## 10. Conclusions

The management of CTEPH should be individualised through case-by-case discussion within a multidisciplinary team at an expert centre, with early referral and, in difficult cases, a second opinion from a high-volume centre before disease is declared inoperable. Riociguat is the medical therapy of choice for inoperable or residual/recurrent disease and is the only agent with Class I randomised support. PH-targeted therapy as a bridge to PEA may be appropriate in selected patients with a high-risk haemodynamic profile, although this rests on expert opinion rather than randomised evidence, whereas riociguat before BPA is supported by randomised data, albeit as a conditional recommendation. When PH persists or recurs after PEA, its mechanism should be defined so that BPA (for residual distal obstruction) and/or PH-targeted therapy (for microvasculopathy) can be directed appropriately. In patients with mixed anatomical lesions, a staged hybrid procedure may be planned on an individual basis, but the supporting evidence is limited and such strategies should not be presented as equivalent to the established treatment arms. Anticoagulation is lifelong, and antiphospholipid syndrome should be sought because it favours vitamin K antagonists over direct oral anticoagulants and may require immunomodulatory therapy. Ongoing randomised trials—including the direct comparison of PEA and BPA and studies of peri-interventional medical therapy—should further define how these modalities are best combined and sequenced. Equally important, earlier recognition through structured follow-up after pulmonary embolism, extended to the wider chronic thromboembolic pulmonary disease spectrum, is needed to bring more patients to these increasingly effective therapies while their disease remains curable.

## Figures and Tables

**Table 1:** Indications, risk modifiers and contraindications, complications, and outcomes of pulmonary endarterectomy (PEA) and balloon pulmonary angioplasty (BPA). Operability and BPA candidacy are determined by multidisciplinary assessment at an expert centre and are not defined by isolated haemodynamic or functional-class thresholds.

	Indications	Risk Modifiers and Contraindications	Complications	Outcome
**PEA**	Surgically accessible thromboembolic obstruction (main, lobar, or proximal segmental arteries) causing pulmonary hypertension Symptomatic disease with an expected haemodynamic benefit that outweighs operative risk Also considered in selected chronic thromboembolic pulmonary disease without resting PH when symptoms are attributable to accessible obstruction Note: operability is a multidisciplinary judgement made at an expert centre; it is not defined by isolated mean PA pressure, PVR, or functional-class thresholds.	Principal true contraindication: severe intrinsic parenchymal lung disease (advanced fibrosis or emphysema), where clearing vessels supplying destroyed lung offers no benefit Anatomical limitation: predominantly distal, surgically inaccessible disease PVR out of proportion to the accessible obstruction (mismatch), predicting residual PH Risk modifiers—not absolute barriers—to be weighed by the team: advanced age, coronary artery disease, left ventricular dysfunction, diabetes, chronic kidney disease; these raise operative risk but do not by themselves exclude a patient from PEA	Neurological complications Persistent pulmonary hypertension Reperfusion lung injury and pulmonary haemorrhage Arrhythmia, atelectasis, wound infection, delirium Operative mortality ~2% at expert centres	Marked and often sustained haemodynamic improvement; potentially curative
**BPA**	Technically inoperable disease (distal or surgically inaccessible lesions) Residual or recurrent PH after PEA Insufficient response to, or contraindication to, surgery	Relative contraindications (most are manageable): iodinated-contrast allergy—usually overcome with premedication (corticosteroid and antihistamine); severe renal dysfunction (contrast-induced injury); active infection; severe hepatic dysfunction; bleeding or clotting tendency; poorly controlled diabetes or hypertension There is no absolute anatomical contraindication; very peripheral disease and long occlusions are technically challenging rather than prohibited at experienced centres	Pulmonary vascular/lung injury (transient desaturation, cough, haemoptysis, severe bleeding, ARDS) Rare vessel perforation Contrast-induced nephropathy Access-site haematoma Medication reactions	Consistent improvement in 6MWD, mPAP, CO/CI, PVR, biomarkers, and functional class across European and Japanese registries [19,22,23,24,25,38,39,40]

PEA candidacy is established by multidisciplinary operability assessment at an experienced centre rather than by isolated haemodynamic or functional-class cut-offs; the comorbidities listed are risk modifiers weighed within that assessment, not automatic exclusion criteria. The principal true contraindication to PEA is severe intrinsic parenchymal lung disease. For BPA, iodinated-contrast allergy is a relative contraindication that can usually be managed with premedication. The 2022 ESC/ERS guidelines define pulmonary hypertension as a resting mean pulmonary artery pressure > 20 mmHg. Bold type in the first column denotes the treatment modality to which the adjacent entries refer. ARDS, acute respiratory distress syndrome; BPA, balloon pulmonary angioplasty; CO/CI, cardiac output/cardiac index; mPAP, mean pulmonary artery pressure; 6MWD, 6 min walk distance; PA, pulmonary artery; PEA, pulmonary endarterectomy; PH, pulmonary hypertension; PVR, pulmonary vascular resistance.

**Table 2:** Factors guiding operability assessment and treatment selection in CTEPH.

Factor	Favours PEA (Operable Disease)	Favours BPA and/or Medical Therapy (Inoperable Disease)
**Lesion location**	Proximal—main, lobar, or proximal segmental arteries	Distal—subsegmental or surgically inaccessible lesions
**PVR–obstruction relationship**	PVR explained by the accessible obstruction	PVR out of proportion to the visible obstruction
**Comorbidity/surgical fitness**	Acceptable operative risk	Prohibitive comorbidity, or patient declines surgery
**Surgical expertise**	Experienced PEA centre available	Lesions beyond surgical reach despite expertise
**Haemodynamic severity**	Operable; very high PVR may prompt medical pre-optimisation	Very high PVR—consider medical pre-treatment before BPA
**Residual PH after PEA**	—	BPA and/or PH-targeted medical therapy

Operability should be judged by a multidisciplinary team at an experienced centre; assessments may differ between centres, and patients should be referred before being declared inoperable. Bold type in the first column denotes the selection factor to which the adjacent entries refer. BPA, balloon pulmonary angioplasty; PEA, pulmonary endarterectomy; PH, pulmonary hypertension; PVR, pulmonary vascular resistance.

**Table 3:** Summary of pivotal randomised controlled trials of medical therapy and of BPA in CTEPH, with trial design and phase. Design and phase are shown to indicate the strength and comparability of the evidence; riociguat (CHEST-1) provides the only Class I, placebo-controlled outcome data.

Trial	Intervention	Design/Phase	Population	Primary Endpoint	Main Result
**CHEST-1** [36]	Riociguat vs. placebo	Phase 3 RCT, placebo-controlled	Inoperable or residual CTEPH	6MWD at 16 weeks	Significant gain in 6MWD; improved PVR, mPAP, FC, NT-proBNP. Basis for approval.
**BENEFiT** [29]	Bosentan vs. placebo	Phase 2 RCT, placebo-controlled	Inoperable or residual CTEPH	PVR and 6MWD at 16 weeks	Improved PVR; no significant change in 6MWD.
**MERIT-1** [31]	Macitentan 10 mg vs. placebo	Phase 2 RCT, placebo-controlled (retracted and republished 2024)	Inoperable CTEPH	PVR at 16 weeks	Improved PVR, 6MWD, NT-proBNP, and cardiac output. Original 2017 report retracted and republished (2024); principal PVR finding preserved.
MACiTEPH [49]	Macitentan 75 mg vs. placebo	Phase 3 RCT, placebo-controlled; stopped early	Inoperable or persistent/recurrent CTEPH	6MWD at 28 weeks	Stopped early for futility; no effect on 6MWD.
**CTREPH** [30]	High- vs. low-dose subcutaneous treprostinil	Phase 3 RCT, active dose comparison (high vs. low dose)	Severe inoperable CTEPH	6MWD at 24 weeks	Higher dose improved 6MWD and PVR; long-term benefit confirmed in OLE [50].
**SELECT** [35]	Selexipag vs. placebo	Phase 3 RCT, placebo-controlled; terminated	Inoperable or persistent/recurrent CTEPH	PVR at 20 weeks	Terminated for futility; no PVR benefit.
Selexipag (Japan) [32]	Selexipag vs. placebo	Phase 2/3 RCT, placebo-controlled (Japan)	Inoperable or persistent/recurrent CTEPH	PVR at 20 weeks	Significant PVR reduction (basis for approval in Japan); mPAP and 6MWD not significantly changed.
MR-BPA [51]	BPA vs. riociguat	Phase 3 RCT, open-label	Inoperable CTEPH	mPAP at 12 months	Greater mPAP reduction with BPA (−16.3 vs. −7.0 mmHg).
**RACE** [21]	BPA vs. riociguat	Phase 3 RCT, open-label	Inoperable CTEPH	% change in PVR at 26 weeks	Greater PVR reduction with BPA; riociguat pre-treatment lowered BPA-related SAEs (14% vs. 42%).

Trials are listed with their phase and design to convey the strength of the underlying evidence; open-label and dose-comparison designs, and trials stopped early, carry less weight than placebo-controlled trials meeting their endpoint. Bold type in the first column denotes the trial name to which the adjacent entries refer. 6MWD, 6 min walk distance; BPA, balloon pulmonary angioplasty; CTEPH, chronic thromboembolic pulmonary hypertension; FC, functional class; mPAP, mean pulmonary artery pressure; NT-proBNP, N-terminal pro-B-type natriuretic peptide; OLE, open-label extension; PVR, pulmonary vascular resistance; RCT, randomised controlled trial; SAE, serious adverse event.

## Data Availability

No new data were created or analysed in this study. Data sharing is not applicable to this article.

## Abbreviations

The following abbreviations are used in this manuscript:

6MWD	6 min walk distance
APS	Antiphospholipid syndrome
ARDS	Acute respiratory distress syndrome
BPA	Balloon pulmonary angioplasty
CMR	Cardiac magnetic resonance
CO/CI	Cardiac output/cardiac index
COPD	Chronic obstructive pulmonary disease
CTEPD	Chronic thromboembolic pulmonary disease
CTEPH	Chronic thromboembolic pulmonary hypertension
DOAC	Direct oral anticoagulant
ECMO	Extracorporeal membrane oxygenation
ERA	Endothelin receptor antagonist
ESC/ERS	European Society of Cardiology/European Respiratory Society
FC	Functional class
IP	Prostacyclin (receptor)
mPAP	Mean pulmonary artery pressure
NT-proBNP	N-terminal pro-B-type natriuretic peptide
NYHA	New York Heart Association
OLE	Open-label extension
PDE5i	Phosphodiesterase-5 inhibitor
PEA	Pulmonary endarterectomy
PH	Pulmonary hypertension
PVR	Pulmonary vascular resistance
RCT	Randomised controlled trial
sGC	Soluble guanylate cyclase
TAPSE	Tricuspid annular plane systolic excursion
UCSD	University of California San Diego
V/Q	Ventilation–perfusion
VKA	Vitamin K antagonist
VTE	Venous thromboembolism
WHO	World Health Organization
WU	Wood units
